# Indicator pathogens, organic matter and LAS detergent removal from wastewater by constructed subsurface wetlands

**DOI:** 10.1186/2052-336X-12-52

**Published:** 2014-02-28

**Authors:** Behrooz Karimi, Mohammad Hassan Ehrampoush, Hossin Jabary

**Affiliations:** 1 Department of Environmental Health Engineering, School of Public Health, Tehran University of Medical Sciences, Tehran, Iran; 2 Department of Environmental Health Engineering, School of Public Health, Shahid Sadoughi University of Medical Sciences, Yazd, Iran; 3 Department of Environmental Health Engineering, School of Public Health, Arak University of Medical Sciences, Arak, Iran

**Keywords:** Wastewater treatment, Pathogens removal, Natural treatment, Constructed wetland

## Abstract

**Background:**

Constructed wetland is one of the natural methods of municipal and industrial wastewater treatments with low initial costs for construction and operation as well as easy maintenance. The main objective of this study is to determine the values of indicator bacteria removal, organic matter, TSS, ammonia and nitrate affecting the wetland removal efficiency.

**Results:**

The average concentration of E. coli and total coliform in the input is 1.127 × 10^14^ and 4.41 × 10^14^ MPN/100 mL that reached 5.03 × 10^12^ and 1.13 × 10^14^ MPN/100 mL by reducing 95.5% and 74.4% in wetland 2. Fecal streptococcus reached from the average 5.88 × 10^14^ in raw wastewater to 9.69 × 10^12^ in the output of wetland 2. Wetland 2 could reduce 1.5 logarithmic units of E. coli. The removal efficiency of TSS for the wetlands is 68.87%, 71.4%, 57.3%, and 66% respectively.

**Conclusions:**

The overall results show that wetlands in which herbs were planted had a high removal efficiency about the indicator pathogens, organic matter, LAS detergent in comparison to a control wetland (without canes) and could improve physicochemical parameters (DO, ammonia, nitrate, electrical conductivity, and pH) of wastewater.

## Background

**Figure 1:**
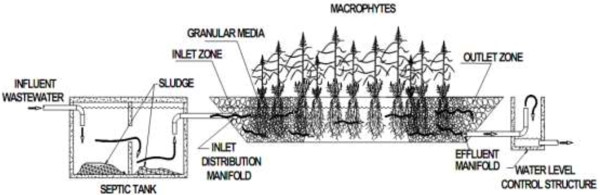
Schematic of subsurface constructed wetland.

Population increase has caused an increasing need to wastewater treatment in many countries. Groundwater contamination to pathogens has recently been prevalent. For example, the prevalence of over 50% diseases in the USA in 2002 was due to the groundwater contamination to wastewater. Lack of wastewater system, pathogen penetration through defective systems of the wastewater treatment and septic tank are considered as the main factors of the groundwater pollution to pathogens [1]. One of the other consequences of industry development is wastewater entry containing constructed organic matter like wastewater contaminated with detergent to the environment [2]. Nearly half a percent of 15 million tons of consumed surfactants in the world in 2001 were synthetic soaps and detergents so that among the synthetic detergents, surfactants of linear alkyl benzene sulfonate (LAS) have had the most production that include about 18% of the total surfactants. LAS have been applied to domestic detergents such as washing powder, dish washing liquid and other domestic detergents [3,4]. Due to increased construction, and maintenance costs as well as the operation of refinery systems, using inexpensive and efficient methods of treatment like constructed subsurface wetland results in water pollution decrease. Constructed wetland is one of the natural methods of municipal and industrial wastewater treatment that considering low initial costs for construction, operation and also very simple maintenance, it was raised as an economical method in engineering plans that can have a desired effect on eliminating environmental pollution [5,6]. High construction and energy consumption costs, the need for complex operation, the need for sludge treatment and disposal, and also the use of automated systems are considered as the major problems of other wastewater treatment methods, but natural systems of wastewater treatment have low technology and yet high efficiency [7,8]. In developed countries, Constructed wetlands are applied to domestic wastewater treatment, agricultural wastewater and runoffs [9,10], industrial wastewater, landfill leachate treatment [11], municipal flood and runoff treatment, advanced clarification and treatment of effluent, regeneration of autotrophic lakes, treatment of contaminated water with nutrients like nitrates [12], as well as phosphates [13], and also effluent denitrification after nitrification performance. Dentrification efficiency of wetlands is dependent on the ratio of C/N. Maximum efficiency is acquired at the ratio of 5:1C/N [8]. A wetland system can treat high levels of chemical and biochemical oxygen demand (COD and BOD_5_), suspended solids (SS), nitrogen and also metals, rare elements, pathogens, constructed organic matter like LAS that is the largest group of anionic surfactants in domestic wastewater [14]. Wetlands have a high biological activity since there are different species of plants, animals and organisms in the soil composition. These conditions will lead to the wastewater treatment and effluent quality improvement [15]. In general, constructed flows are classified into surface flow wetlands with free water surface (FWS) and subsurface flow (SSF). Wastewater flow in subsurface flow (SSF) can be made as the vertical upward flow and horizontal one. A wetland is filled with sand and soil with appropriate aggregation. This bed will create a suitable surface for growth of microbes [16]. A scheme of constructed subsurface wetland and LAS chemical formula are available in Figure 1. The purpose of this study is to consider removal values of indicator bacteria (total coliform, Fecal coliform, and streptococcus) and also to consider removal values of linear detergents (LAS), organic matter, TSS, ammonia, nitrate, and DO. The study of these parameters will also be necessary due to the effect of general parameters such as temperature, pH, and electrical conductivity in the growth of plants and bacteria reproduction.

## Methods

### The characteristic of wastewater

The average of different parameters in raw wastewater before entering the wetlands is as follows: the flow of input wastewater to each wetland 14 m^3^/day, average temperature of the input 15.95°C, electrical conductivity 1714 μS/cm, dissolved oxygen 0 mg/L, the value of BOD_5_ 176.6 mg/L, ammonia 110 mg/L, the value of COD 385.6 mg/L, LAS 10.65 mg/L and pH is 7.7.

### The wetlands systems

The study method is descriptive-analytical, and the studied society is Yazd municipal wastewater. Raw wastewater was entered into the septic tank after passing a preliminary treatment and got into four subsurface wetlands (SSF) with three different species of canes. One of the ponds was taken into consideration as the control wetland without planting any plants. Overall dimensions of the wetlands are 90 m^2^ with a hydraulic retention time of 3–5 days. The bed was filled with an effective size of gravels 0.2 to 1 cm and the height of 30 cm; at the beginning of the input and in the output, sands with coarse size of 10 cm were used. The number of canes per area unit was 1000 canes per square meter considered the same for all three wetlands. After planting the canes and their enough growth, continuous samplings of the output effluent were taken to reach a steady state; then after six months, the wetlands reached the desired condition. Constructed wetlands were designed so that surface runoffs do not get into them. No plants were planted in any of these four wetlands, and filling materials were applied like other beds (control wetland), and in other wetlands, four different species of canes from Phragmites, Phalaris and Glyceria family with local names of Bafgh, Aliabad, and Yazdbaf species were planted that in the paper they have been named with the wetland 1, 2, and 3. Entering the wastewater into the beds, consecutive samplings were conducted from all wetlands so as to reach a stable condition. After 28 days, all wetlands reached the stable condition, and the study began. Sampling points are available in the output of each wetland and before the entry. All tests at each stage were repeated five times and the average removal efficiency of each parameter was obtained.

### Chemicals

The iron sulfate (FeSO_4_.7H_2_O), H_2_O_2_ (30% W/V), H_2_SO_4_, NaOH, acetic acid (CH_3_COOH), potassium dichromate (K_2_Cr_2_O_7_), HgSO_4_, Ag_2_SO_4_, manganese oxide and powder and granular activated carbon were purchased from Merck, Germany.

### Instrumentation and analysis method

An analysis method of membrane filtration is carried out using membrane filter 0.45 μm and a propitiatory medium of each of the above bacteria which is available in Table 1[17]. In order to consider the microbial quality, 0.5 liter of the sample was placed in a sterile glass container; its cap was closed quickly, and was located adjacent to ice. To conduct other chemical parameters, two liters of the sample were taken using a polyethylene container to keep and carry the sample. About 100 samples were taken during six months and factors, including biochemical oxygen demand, total coliform, fecal coliform, streptococci, TSS, EC, pH, DO, temperature, etc. were measured. Experiments related to the concentration of ionic surfactants of LAS were conducted by MBAS (5540.C method). Figure 1 showed schematic of subsurface constructed wetland. All sampling conditions and experiments have been carried out based on guidelines of Standard Method [17]. To determine the concentration of ammonia, a device DR/5000 was applied [9]. Analysis methods of the output sample are available in Table 1.

**Table 1:** Samples Analysis methods

**Parameter**	**Method**	**Sample size and type of sample container**	**Sample holding time**	**Conservation methods**	**References**
Fecal Coliforms	9222 E	150 ml, Sterile glass vessel	6 h	Cooling ،4°C	APHA-AWWA،1995
E. Coli	9260 F	150 ml, Sterile glass vessel	6 h	Cooling ،4°C	APHA-AWWA،1995
Fecal streptococci	A 9230	150 ml, Sterile glass vessel	6 h	Cooling ،4°C	APHA-AWWA،1995
TSS	D 240	500 ml, Sterile glass or Polyethylene vessel	7 day	Cooling ،4°C	APHA-AWWA،1995
BOD_5_	405.1	1000 ml, Sterile glass or Polyethylene vessel	48 h	Cooling ،4°C	USEPA 1983
NH_4_^+^	350.3	400 ml, Sterile glass or Polyethylene vessel	28 day	Cooling ،4°C, 2 cc Sulfuric acid	USEPA 1983
LAS	5540C	1000 ml, Sterile glass or Polyethylene vessel	48 h	Cooling ،4°C	APHA-AWWA،1995

The examiner's name, date and time of sampling, and other specifications, including weather conditions were written carefully on each sample. Specifications of four wastewater treatment systems are available in Table 2.

**Table 2:** Characteristic of 4 constructed pilot systems for waste water treatment

**Parameter**	**Site 1**	**Site 2**	**Site 3**	**Control**
wastewater	Domestic + Septic Tank	Domestic + Septic Tank	Domestic + Septic Tank	Domestic + Septic Tank
Bed dimensions (m)	5 × 4.5 × 0.7	5 × 4.5 × 0.7	5 × 4.5 × 0.7	5 × 4.5 × 0.7
Bed surface area (m^2^)	22.5	22.5	22.5	22.5
Type of flow	horizontal	horizontal	horizontal	horizontal
Hydraulic loading (m^3^/(m^2^ h)	0.04	0.04	0.04	0.04
Flow rate m^3^/h	20	20	20	20
Hydraulic retention time (d)	3-5	3-5	3-5	3-5
Type of reed	Phragmites	Phalaris	Glyceria	No plant

## Results

There are uniform changes in the physical parameters of wastewater (pH, temperature, EC, and DO). Dissolved oxygen values from 0 in the input to the average 1.6 ± 0.78 mg/L in the wetland 2 indicate an increasing trend of this parameter and a reduction in organic load values of the input. The removal efficiency of TSS for the wetlands is 68.87%, 71.4%, 57.3%, and 66% respectively. In Table 3, there is the average input and output parameters of four studied wetlands.

**Table 3:** Comparison of average input and output parameters of 4 wetland systems

**Parameter**	**Inputs**	**Site 1**	**Site 2**	**Site 3**	**Control**
Fecal Coliforms	4.41 × 10^14^	1.14 × 10^14^	1.13 × 10^14^	7.84 ×10^12^	1.1 ×10^14^
E. Coli	1.127 × 10^14^	1.1 × 10^14^	5.03 × 10^12^	2.44 × 10^11^	1.31 × 10^12^
Fecal streptococci	5.88 × 10^14^	1.55 × 10^13^	1.16 × 10^14^	9.69 × 10^12^	3.34 ×10^12^
TSS (mg/L)	102.8 ± 42.6	32 ±18.86	29.4 ± 12.68	43.9 ± 24	34.9 ±19.2
Range	1-156	3-62	2-51	14-73	3-119
NH_4_^+^ (mg/L)	110 ± 51.6	129.7 ± 36.48	121 ± 30.16	127.2 ± 27.37	112.2 ± 38.42
Range	15-22	70-207	90-185	95-196	45-182
NO3 (mg/L)	15.4 ± 8.15	17.63 ± 9.6	14.42 ± 8.85	18 ± 11.4	16.85 ± 13.2
Range	2.8-25.4	4-33.6	0-28.5	0-31.7	0-35.8
DO (mg/L)	0	1.47 ± 0.47	1.6 ± 0.57	1.59 ± 0.43	1.75 ± 0.78
Range	0	0.91-2.5	1.05-2.8	1.24-2.5	1.25-3
PH	7.7 ± 0.13	7.88 ± 0.13	8 ± 0.11	7.9 ± 0.15	8.05 ± 0.155
Range	7.88-7.49	7.61-8.05	7.76-8.09	7.62-8.17	7.49-8.27
EC	1715 ± 425.5	2339.4 ± 319	2375.2 ± 603.75	2286.3 ± 370	2044.7 ± 327.5
Range	1052-2460	1994-2960	1574-3550	1793-3050	1247-2470
COD (mg/L)	385.5 ± 15	130 ± 26.4	102 ± 19.3	106 ± 22.4	92.5 ± 6.4
Range	250-730	80-160	80-150	65-140	55-130
BOD5 (mg/L)	176.6 ± 2.4	45 ± 14.2	41 ± 21	46.5 ± 16	37.5 ± 3.9
Range	103-250	15.5-74	27-61	26-77	17-58
LAS (mg/L)	10.6 ± 0.8	2.1 ± 0.8	2.8 ± 0.5	2.4 ± 0.7	3.1 ± 1.9
Range	5.7-15.6	1.5-2.5	1.8-2.9	1.8-2.5	2.5-3.7

**Figure 2:**
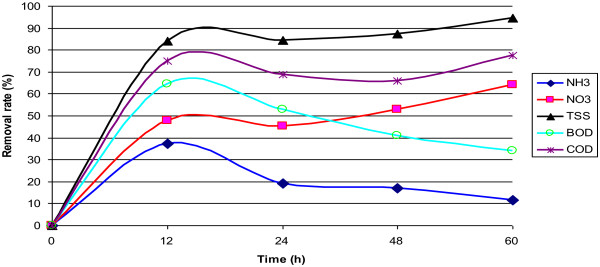
Removal of ammonia, nitrate, suspended solids, BOD and COD in Site 1.

**Figure 3:**
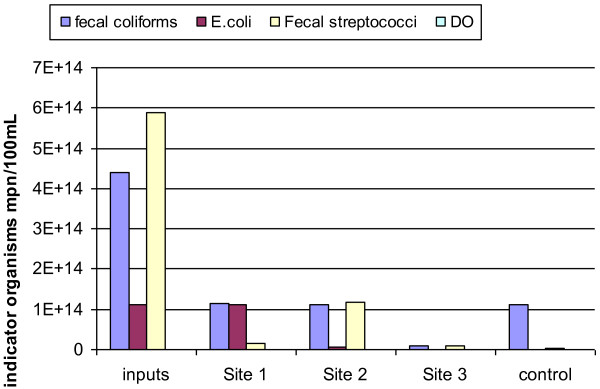
Effect of dissolved oxygen on the indicator organisms in raw wastewater and output 4 synthetic wetlands.

**Figure 4:**
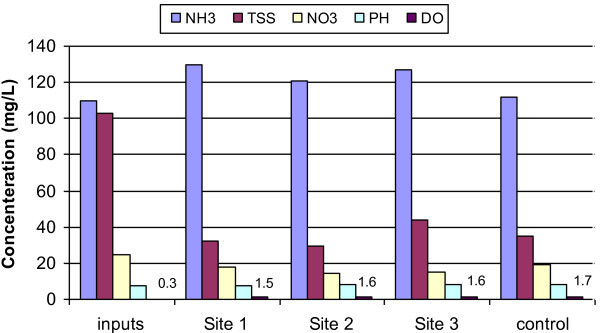
Effect of dissolved oxygen on nitrate and ammonia reduction in raw wastewater and output 4 synthetic wetlands.

In Figure 2, the removal efficiency of ammonia, nitrate, suspended solids, BOD and COD is available in site 1. The effect of dissolved oxygen on indicator organisms is available in Figure 3, and the effect of dissolved oxygen on ammonia and nitrate in raw wastewater and the output of four constructed wetlands are available in Figure 4.

The average concentration of E. coli and the total coliforms in the input are 1.127 × 10^14^ MPN/100 mL and 4.41 × 10^14^ MPN/100 mL that reached 5.03 × 10^12^ MPN/100 mL and 1.13 × 10^14^ MPN/100 mL by reducing to 95.5% and 74.4% in wetland 2; while the concentration of fecal streptococcus reached from the average 5.88 × 10^14^ MPN/100 mL in the input wastewater to 1.55 × 10^13^ MPN/100 mL, 1.16 × 10^14^ MPN/100 mL, 9.69 × 10^12^ MPN/100 mL vand 3.34 × 10^12^ MPN/100 mL respectively in the output of each wetland 1, 2, 3 and control. In general, wetland 2 could reduce 1.5 logarithmic units of E. coli. The E. coli concentration was different in the output effluent of each wetland and included from the concentration of 1.1 × 10^15^ to 8.5 × 10^1^ MPN/100 mL. Regression coefficient was 0.77 for E. coli (Table 4). The maximum removal value of Escherichia coli is concerned with wetland 2 with 95.5% efficiency.

**Table 4:** Pearson correlation between the frequency of microorganisms and physico-chemical parameters in the system

**parameter**	**NH**_**4**_^**+**^**-N**	**TSS**	**pH**	**EC**	**TC**	**E. coli**	**DO**
NO_3_^+^	0.45 (**)	−0.053	0.082	0.04	0.022	0.064	−0.18
TSS	0.04	1	−0.514 (**)	−0.458 (**)	0.257	0.069	−0.47 (**)
PH	0.17	−0.5 (**)	1	0.28 (*)	−0.136	−0.154	0.45 (**)
EC	0.096	−0.46 (**)	0.287 (*)	1	−0.034	−0.07	0.25
TC	(*) 0.286	0.257	−0.136	−0.034	1	−0.52 (**)	−0.416 (**)
FS	0.166	0.537 (**)	−0.465 (**)	−0.198	0.7 (**)	0.224	−0.55 (**)

N-NH: ammonium; TC: total coliforms; FC: fecal coliforms; FS: fecal streptococcus; DO: dissolved oxygen.

**Correlation is significant at the 0.01 level (2-tailed).

*Correlation is significant at the 0.05 level (2-tailed).

In the present study, the removal percentage of detergents, COD, and BOD are respectively equal to 80 - 95%, 61 - 85%, and 62 - 96% that is a relatively desired efficiency. The average value of input LAS to the system 10.65 mg/L; the average value of output LAS from the system 1.9 mg/L; and thus the overall system efficiency in removing detergents is 82%. The ammonia concentration has reached from the average input value 110 to 129.7 mg/L at site 1 and to 127.1 mg/L at site 3 so that we will witness less amounts of ammonia in the control wetland and site 2 with less coverage of canes.

## Discussion

The trend of changes in the solids in the total study period expresses a dramatic difference in the input compared to the output of each of the four wetlands. The statistical results show the reduction of suspended solids along with reducing indicator organisms. For example, fecal streptococcus of Pearson's correlation test with a significant level of 0.01 was P_value_ = 0.5 expressing a positive relationship between suspended solids and Fecal streptococcus. A statistical analysis of (Kruskal Wallis Test) expressed a significant statistical difference in removing total coliforms P_value_ = 0.05 streptococcus P_value_ = 0.019 in the constructed subsurface wetland (Table 5).

**Table 5:** Kruskal Wallis Test Analysis between different groups of microorganisms

**Parameter**	**Total coliforms**	**E. coli**	**Fecal streptococcus**
Chi-Square	9.4	5.8	11.7
df	4	4	4
Asymp. Sig.	0.05	0.214	0.019

The removal of total and fecal coliforms is caused by biological factors like hunter organisms such as nematodes, protozoa, bacterial activity, bacteriophage production, chemical factors, like oxidation reactions, bacterial uptake and toxicity, as well as plant absorption. Generally, factors that result in the removal of coliforms in a wetland include sunlight, the presence of predators, bacteriophages, lack of nutrients, and rare elements, toxicity of other microorganisms, etc. [18]. Sedimentation process is also effective in removing coliforms and Fecal streptococci [19,20]. Filtration and microbes clinging to the root level are other methods of organism reduction, but it is possible that microbes clinging to the root of plants lead to the reduction of sedimentation of microbes and viruses in wetlands [21]. As it was seen in the results of this study, the removal of bacteria in the wetland is very much and is related to the removal of suspended solids. The increased removal of suspended solids will give rise to the increased removal of bacteria. Direct feeding of protozoa from E. coli in wetlands has been proved. The hunt for and natural mortality of pathogens like Escherichia coli and cryptosporidium are also existed in wetlands [22]. In addition, temperature reduction can have a direct effect on the growth of E. coli. In a study conducted by Decamp et al., 2000, the average removal of E. coli was 41 -72% on the actual scale and 96.6 - 98.9% on the pilot scale. Reduction of the retention time decreased the pilot system efficiency [23]. In a study performed by Evanson et al., 2006, the removal value of fecal coliforms is 82.7% - 99.95%. An analysis of T-tests indicated that there is not a statistical relationship between the input and output (P < 0.01). Moreover, the removal value of TSS was between 25% - 89.1%, and on average, the removal efficiency of suspended solids was reported 55.8 ± 52.8% [24]. Processes that are performed by microorganisms, like nitrification (in aerobic conditions) and dentrification (in anaerobic conditions) interfere in the control and removal of nitrogenous compounds. Chemical precipitation and absorption of nutrients like phosphate is performed by soil particles. Drastic changes in the efficiency of wetlands can be resulted from climate changes, sunlight intensity and weakness, water depth, etc. [25]. Microorganisms are trapped by above mechanisms in the wetland and due to the stop and longevity and food reduction, they will get into the demolition phase [26].

Given the numbers obtained from this research, the most removal value for LAS is concerned with cane species of Yazd (Phragmites, Site 1) with the removal efficiency of 81.6% and following that the cane of Aliabad (Phalaris, Site 2) with overall efficiency of 80% and the removal efficiency for Bafgh (Glyceria, Site 3) is 80%. The removal percentage of septic tank is 31.45% for this parameter indicating the low efficiency of anaerobic system for removing LAS. Figure 3 was show LAS ultimate Biodegradation and mineralization. In a control wetland, the output value is specifically high. In a study conducted by Amirmozafari et al., 2007 on separating ionic surfactants, they concluded that ionic detergents have a cumulative property in domestic and industrial wastewater and due to foam formation; they can cause direct toxic effect on some organisms [27]. In addition, the more amount of ammonia is added; the amount of nitrate is lessened.

In four wetlands, nitrate is also reduced proportionally. Statistical results indicate no significant relationship between the input and output nitrate. Dissolved oxygen values strongly affect the efficiency of wetlands. With increasing dissolved oxygen values, the amount of organisms are lessened, and there is a direct statistical relationship between the increase of DO with indicator organisms so that the correlation coefficient for total coliforms is 0.416 and for Fecal streptococci is −0.555 (with a significant level 0.01), whereby the negative sign indicates an indicator organism with increasing the dissolved oxygen [28].

## Conclusion

The study considers the subsurface wetlands process to reduce indicator pathogen and organic load, TSS, ammonia, nitrate, and DO from the waste water that pretreated by septic tank. The overall results show that wetlands in which herbs were planted had a high removal efficiency about the indicator pathogens, organic matter, LAS detergent in comparison to a control wetland (without canes) and could improve physicochemical parameters (DO, ammonia, nitrate, electrical conductivity, and pH) of wastewater. The advantages of this method (wetland) compared to other ones are simple performance, the use of indigenous and natural canes at a building site, low cost of construction, lack of insects' accumulation (subsurface flow), lack of production of unpleasant smell, lack of creation of a beautiful green space, lack of growth of mosquitoes, lower level of a required land, an appropriate place to attract wildlife (birds, reptiles, etc.), and the disadvantages of this method include bed obstruction, increased costs of cleaning, etc.

## Competing interests

The authors declare that they have no competing interests.

## Authors’ contributions

This work is part of the Master thesis of BK that MHE developed initial idea and proposed and supervised the whole work. BK administered data collection. All authors read and approved the final manuscript.
